# Progesterone levels on the day of embryo transfer using a single pessary of 400mg of vaginal progesterone vs. 200mg x2 pessaries in hormonal replacement cycles

**DOI:** 10.5935/1518-0557.20230021

**Published:** 2023

**Authors:** Alicia Herencia, Andrea Bernabeu, Anna Pitas, Jose Antonio Ortiz, Cristina Gavilán, Sonia Albero, Juan Carlos Castillo, Rafael Bernabeu

**Affiliations:** 1 Instituto Bernabeu Madrid. Ayala 48. 28001, Madrid, Spain; 2 Instituto Bernabeu Alicante. Albufereta 31.03016, Alicante, Spain; 3 Instituto Bernabeu Mallorca. Aragó 8. 07006, Palma, Islas Baleares, Spain

**Keywords:** embryo transfer, luteal phase support, miscarriage, pregnancy, progesterone

## Abstract

**Objective:**

Does the use of 400mg pessaries of micronized progesterone provide comparable results as pessaries of 200mg x2, in terms of progesterone levels in hormonal replacement cycles for embryo transfer?.

**Methods:**

Retrospective cohort study based on 299 embryo transfer treatments under artificial endometrial preparation carried out at Instituto Bernabeu. 131 patients received 1 pessary of 400 mg b.i.d. (group A) and 168 received 2 pessaries of 200 mg b.i.d. (group B).

**Results:**

Mean serum progesterone levels were similar between groups (A: 13.64±4.47ng/mL *vs*. B: 13.88±7.17ng/mL). There were no differences in suboptimal progesterone levels between groups (A: 11.5% vs. B: 16.8%). In terms of patients receiving additional progesterone supplementation, there were no differences between groups (A: 26% *vs*. B: 35.3%.). No differences between groups were observed in clinical outcomes: pregnancy rate (PR) (A: 55% *vs*. B: 54.8%), biochemical pregnancy loss rate (BPLR) (A: 13.4% *vs*. B: 17.6%), miscarriage rate (MR) (A: 17.9% *vs*. B: 19.8%) and ongoing pregnancy rate (OPR) (A: 36.5% *vs*. B: 34.1%).

**Conclusions:**

One progesterone pessary of 400mg (Cyclogest^®^) twice daily appears to be non-inferior to the use of two-200mg pessaries twice daily in terms of progesterone levels in HRT cycles.

## INTRODUCTION

Cryopreservation techniques have evolved over the years, mainly after embryo vitrification was consistently introduced. Frozen-thawed embryo transfer (FET) cycles are currently a widely used technique in IVF treatments (Toner *et al*., 2016). The most frequently protocol for this purpose is artificial supplementation with estrogens and progestins, known as hormonal replacement therapy (HRT). Several alternatives are available to perform luteal phase supplementation in HRT cycles aiming to stage and synchronize the uterine lining for embryo implantation and subsequent maintenance of the pregnancy during first trimester. Vaginal delivery is the preferred route of progesterone administration in Europe. Its effectiveness has been proved, either because of its capacity to induce decidualization in an estrogen-stimulated endometrium, or due to the potential to supplement luteal phase in ART. However, in HRT cycles, there is concern over vaginal progesterone administration not reaching appropriate serum levels, which might lead to treatment failure and subsequent implantation failure or eventual miscarriage (Labarta *et al*., 2017).

Across Europe, 200mg micronized vaginal progesterone (Utrogestan^®^, Progeffik^®^) has been extensively used aiming to stage and synchronize the uterine lining, being two vaginal pessaries twice a day (800mg/d in total) one of the most accepted protocols (Labarta *et al*., 2017). Recently, the European Medicines Agency has authorized commercialization of 400mg micronized vaginal progesterone pessaries (Cyclogest^®^). The reduction of vaginal application would lead to better therapy compliance and less side effects and discomfort for the patients. This brand-new preparation has been compared to other luteal phase support methods in fresh IVF cycles, but so far, comparative studies are lacking when it comes to HRT endometrial preparation (Saunders *et al*., 2020). Due to this evidence gap, our study aims to determine whether the administration of one 400mg micronized vaginal progesterone pessary administered every 12 hours is non-inferior to two 200mg micronized vaginal progesterone pessaries every 12 hours in terms of progesterone serum levels in patients undergoing blastocyst transfer following artificial endometrial preparation.

## MATERIALS AND METHODS

### Study design

This retrospective cohort study was carried out at Instituto Bernabeu between January 2019 and July 2020. We included 299 frozen embryo transfers performed under HRT, 241 of them were embryos coming from oocyte donation cycles. Patients were included only once and were allocated in two groups depending on the vaginal micronized progesterone regime employed.

Main inclusion criteria were as follows: women between 18-50 years diagnosed with primary or secondary infertility subduing endometrial preparation with HRT for embryo transfer; normal uterine cavity; blastocyst stage embryo transfers on day 5 and documented progesterone serum levels on the day of the embryo transfer. Exclusion criteria included patients having a different type of progesterone regimen supplementation, significant uterine conditions (e.g. fibroids, polyps, Müllerian abnormalities, Asherman syndrome), the presence of hydrosalpinx or endometritis.

Additional patient characteristics such as age, body mass index (BMI), weight, previous down-regulation with gonadotrophin-releasing hormone (GnRH) agonist or menopausal status were also registered in our database.

### Study endpoints

The main study endpoint was progesterone serum levels in patients undergoing embryo transfers in HRT cycles measured after 5 days of progesterone administration.

Secondary endpoints included the incidence of suboptimal progesterone levels (defined as below 8.8ng/mL) (Labarta *et al*., 2021); the rate of patients who were supplemented with additional subcutaneous progesterone; pregnancy rate (PR); biochemical pregnancy loss rate (BPLR), miscarriage rate (MR); ongoing pregnancy rate (OPR).

### Study protocol

Hormone replacement therapy was started on day 1-3 of menstruation, with either 6mg/day of estradiol valerate orally, or transdermally (150µg of estradiol hemihydrate). After 10-12 days on estrogens, a vaginal scan ultrasound was performed to assess endometrial thickness. Patients were divided in two groups depending on vaginal micronized progesterone regimen: patients receiving one pessary of 400mg (Cyclogest^®^) twice daily (Group A) and patients receiving 2 pessaries of 200mg (Utrogestan^®^ or Progeffik^®^) twice daily (Group B). Progesterone levels were assessed the day of the embryo transfer by an electrochemiluminescence immunoassay (Cobas^®^ e 411 analyzer, Roche diagnostics GmbH, Germany). Samples were obtained 2-5 hours after the last progesterone administration. We considered 8.8ng/mL as the cut-off-value to define low progesterone levels, as previously reported (Labarta *et al*., 2021); thus, results below that cut-off-value were deemed as suboptimal progesterone levels.

All embryo transfer procedures were performed at blastocyst stage following 5 days of progesterone administration. Embryo quality was graded according to ASEBIR (Cuevas Saiz *et al*., 2018).

Additional data was obtained from a sub-group of patients who received extra subcutaneous progesterone despite serum progesterone levels being optimal (over 8.8ng/mL), based on clinician’s decision (mainly because some other medical criteria such as previous low progesterone levels or previous early pregnancy losses).

### Statistical analyses and sample size calculation

Data coming from a recent prospective study employing a similar HRT for endometrial preparation and progesterone supplementation with 200mg x 2 vaginal capsules (Utrogestan^®^) twice daily, demonstrated a mean progesterone level of 12.7±5.4ng/mL (Labarta *et al*., 2017). Based on this data and accepting an alpha risk of 0.05 and a beta risk of 0.20 in a unilateral contrast (statistical power of 80%), a sample size of 182 patients (91 in each arm) were required to detect a minimum difference of 2ng/mL. Assuming a loss rate of 10%, at least 200 patients (100 in each arm) were established as an adequate sample size.

The comparison between the study groups for categorical variables was performed using Chi-square test. The normal distribution of the variables was analyzed using the Shapiro-Wilk test. If the distribution was normal, the comparison between the different groups was carried out using t-Student, and otherwise the Mann Whitney U-test.

To analyze the influence of patient characteristics on serum progesterone on the day of ET, multivariate logistic regression analysis was performed. Variables that were correlated in a univariable analysis with serum progesterone levels were included.

Differences were considered statistically significant when *p*<0.05.

## RESULTS

### Patient demographics and baseline characteristics

For luteal phase supplementation, 131 patients were included in Group A and 168 in Group B. Overall, mean age was 41.45 years, and mean weight and BMI were 64.41 and 23.59 kg, respectively; 90.2% was Caucasian population.

Among women included in the analysis, 52.2% received previous down-regulation with GnRH agonist. Mean number of transferred embryos was 1.09, of which, 96% were good quality embryos.

Both groups were comparable in terms of number of embryos transferred (Group A: 1.12±0.323 *vs*. Group B: 1.07±0.259, *p*=0.147). Differences were found between both groups in terms of weight (Group A: 66.77±14.11 *vs*. Group B: 61.88±11.88, *p*=0.002) and BMI (Group A: 24.68±4.99 *vs*. Group B: 22.60±4.31, *p*<0.001).

### Outcomes

The main study outcomes are shown in Table 1. Mean progesterone levels were similar in both groups (Group A: 13.64ng/mL±4.47; Group B: 13.88ng/mL±7.17; *p*=0.325).

**Table 1:** Differences in progesterone levels between both groups. The comparison between the study groups for categorical variables was performed using Chi-square test. The normal distribution of the variables was analyzed using the Shapiro-Wilk test. If the distribution was normal, the comparison between the different groups was carried out using *t*-Student, and otherwise the Mann Whitney U-test.

	Cyclogest^®^ 400/12h Group A	Natural micronized progesterone 200mg x2/12h Group B	*p*
**Mean Progesterone Levels**	13.64±4.47ng/mL	13.88±7.17ng/mL	0.325
**Additional Subcutaneous Progesterone**	26.0%	35.3%	0.083

The incidence of suboptimal levels of progesterone (below 8.8ng/mL) was similar in both groups (11.5% in Group A *vs*. 16.8% in the Group B; *p*=0.195). (OR 1.76; 95%CI, 0.85-3.67) (Table 2).

**Table 2:** Rate of low progesterone serum levels.

	Cyclogest^®^ 400/12h Group A	Natural micronized progesterone 200mg x2/12h Group B	OR	IC
Progesterone<8.8 ng	11.5%	16.8%	1.76	0.85-3.67

In the sub-group of patients receiving extra subcutaneous progesterone from the day of the embryo transfer despite having normal serum progesterone levels (based on clinician´s decision); the frequency of additional supplementation was also similar, although again lower in the group receiving Cyclogest^®^ 400 (26% in Group A *vs*. 35.3% in the Group B; *p*=0.083) (Table 1).

Reproductive outcomes were similar between Group A and Group B in terms of PR (55% *vs*. 54.8%, *p*=0.973), BPLR (13.4% *vs*. 17.6%, *p*=0.480), MR (17.9% *vs*. 19.8%, *p*=0.767) and OPR (36.5% *vs*. 34.1%, *p*=0.673) (Table 3).

**Table 3:** Reproductive outcomes. (PR=pregnancy rate; BPLR=biochemical pregnancy loss rate; MR=miscarriage rate; OPR=ongoing pregnancy rate; BMI=body mass index). To analyze the influence of patient characteristics on serum progesterone on the day of ET, multivariate logistic regression analysis was performed.

	Cyclogest^®^ 400/12h Group A	Natural micronized progesterone 200mg x2/12h Group B	*p*
**PR**	**55.0%**	**54.8%**	**0.973**
**BPLR**	**13.4%**	**17.6%**	**0.480**
**MR**	**17.9%**	**19.8%**	**0.767**
**POR**	**36.5%**	**34.1%**	**0.673**
**Body Weight**	**66.77±14.11**	**61.88±11.88**	**0.002**
**BMI**	**24.68±4.99**	**22.60±4.31**	**<0.001**

## DISCUSSION

To the best of our knowledge, this is the first study comparing the progesterone levels on the day of embryo transfer with 200mg x 2 *vs*. 400mg twice daily in blastocyst stage HRT cycles. Our results indicate that both regimens resulted in similar progesterone levels when measured after five days of progesterone administration. In further support of a similar luteal phase environment, the pregnancy rates for the two groups were also similar.

Overall, in ART cycles, administering progesterone for replacement therapy has been challenging. With the oral route, > 90% of progesterone is metabolized by the liver at first hepatic pass, and the metabolites are associated with dizziness and drowsiness. Although synthetic progestins have been developed to resist degradation, these compounds are commonly avoided over concerns of teratogenesis. In the USA, intramuscular P is also frequently used; however, this route is generally not preferred by patients due to pain associated with injections. Vaginal delivery is the preferred route of P administration in Europe and the vaginal route has been used successfully in donor egg replacement cycles as well as HRT-FET cycles (Toner, 2000). Recently, in HRT cycles, luteal phase supplementation using vaginal natural micronized progesterone is a topic of increasingly research (Labarta *et al*., 2021). Even though the vaginal route allows rapid absorption of progesterone across the epithelium and achieves a high concentration of progesterone in endometrial tissue while limiting systemic exposure (Paulson *et al*., 2014) a significant inter-individual variability is constantly reported when measuring progesterone or other hormone levels, which might be the consequence of variations in the vaginal absorption rate or bioavailability (Labarta *et al*., 2021). Moreover, recent publications shows that a minimum level of serum P is required to optimize clinical outcome suggesting that inadequate levels of progesterone are associated with poorer outcomes (Labarta *et al*., 2017; 2021; Yovich *et al*., 2015; Cédrin-Durnerin *et al*., 2019; Gaggiotti-Marre *et al*., 2019). Thus, additional research is needed to determine which is the best dose for each patient, taking into consideration individual patient characteristics. Recent observational study points some of this individual patient factors associated with low progesterone levels are BMI, parity and non-European geographic origin (Maignien *et al*., 2022).

Acknowledging the inherent biases to a retrospective design, and the differences in terms of weight and BMI between both groups, our results show that the use of one pessary of 400mg of natural micronized progesterone (Cyclogest® 400) administered twice daily provides equivalent mean progesterone levels when compared to the use of two-200mg of vaginal capsules twice daily in HRT cycles. Moreover, even though the study was not powered to detect differences in pregnancy rates and the fact that patients received extra doses of progesterone at clinician´s choice, the achievement of similar pregnancy rates suggests that -overall-, a similar optimal luteal phase support in HRT cycles can be expected with either approach. However, when using the vaginal route for progesterone administration, previous studies in HRT cycles have suggested that vaginal progesterone itself, the pharmaceutical vehicle, or fluctuations in other endogenous hormone concentrations, may influence the vaginal tissue with respect to progesterone absorption (Paulson *et al*., 2014). In this regard, Cyclogest® provides similar frequency of suboptimal levels of progesterone (<8.8ng/mL), suggesting the ability from this formulation in achieving sustained progesterone levels in HRT cycles. As previously noted, this point is of valuable importance in view of the cumulative evidence suggesting a critical threshold of serum P around 9ng/mL for an optimal luteal phase support with progesterone in HRT cycles. Patients exhibiting lower levels shows a significantly lower ongoing pregnancy rate and higher miscarriage rate as demonstrated in two prospective clinical trials (Labarta *et al*., 2017; 2021) and “rescue” strategies with additional subcutaneous or intramuscular progesterone have been suggested when these values are observed. In this line, a potential clinical implication of our findings suggest that “rescue” strategies with additional progesterone would be less required in HRT cycles supplemented with Cyclogest^®^ 400mg twice daily. Further studies are needed to support these initial findings.

In conclusion, the administration of one pessary of 400mg of natural micronized progesterone (Cyclogest^®^ 400) twice daily appears to be non-inferior to the use of two-200mg of vaginal capsules twice daily in terms of progesterone levels in HRT cycles when measured after five days of progesterone administration. Moreover, Cyclogest^®^ 400mg administration might reduce the frequency of suboptimal progesterone levels on the day of embryo transfer compared to the use of two-200mg of vaginal capsules. Cyclogest^®^ 400 approach might have the clinical implication of minimizing the need for additional progesterone administration or “rescue” strategies.

The findings of our study should be corroborated with larger adequately powered dose finding and pharmacokinetic studies to confirm or refute a comparable effect of Cyclogest^®^ 400 *vs*. vaginal capsules, particularly on the impact on the live birth rate and the need for “rescue” strategies. If confirmed, these findings would represent a step towards the optimization of luteal phase support using vaginal progesterone in HRT cycles.
